# Risk factors related to low-level viraemia in chronic hepatitis B patients receiving entecavir treatment

**DOI:** 10.3389/fcimb.2024.1413589

**Published:** 2024-08-07

**Authors:** Zhong-Bin Li, Dan-Dan Chen, Yun-Fei Jia, Qing-Juan He, Li Cui, Feng-Xia Du, Yao-Jie Kang, Xin Feng, Mengwen He, Xue-Yuan Jin, Jing Chen, Yudong Wang, Dong Ji, George Lau, Shu-Gao Wu

**Affiliations:** ^1^ Senior Department of Hepatology, the Fifth Medical Center of PLA General Hospital, Beijing, China; ^2^ Department II of Infectious Diseases (Hepatology), The Second People’s Hospital of Jingzhou City, Jingzhou, China; ^3^ Department of Hepatobiliary & Gastrointestinal, Henan Province Hospital of Traditional Chinese Medicine, Zhengzhou, China; ^4^ Department II of Gastroenterology, The Eighth People’s Hospital of Qingdao, Qingdao, China; ^5^ Department of Emergency, the Fifth Medical Center of PLA General Hospital, Beijing, China; ^6^ Department of Pharmacy, Medical Supplies Center of PLA General Hospital, Beijing, China; ^7^ Department of Medical Quality Management, the Fifth Medical Center of PLA General Hospital, Beijing, China; ^8^ Out-patient Department, Hospital of Beijing Information Science and Technology University, Beijing, China; ^9^ 302 Clinical Medical School, Peking University, Beijing, China; ^10^ JCSchool of Public Health and Primary Care, Chinese University of Hong Kong, Hong Kong, Hong Kong SAR, China; ^11^ Humanity and Health Clinical Trial Center, Humanity and Health Medical Group, Hong Kong, Hong Kong SAR, China

**Keywords:** low-level viremia, effectiveness, nucleos(t)ide analogs, complete virological response, high-risk factor

## Abstract

**Background:**

About 20% of on-treatment patients with chronic hepatitis B (CHB) experienced low-level viraemia (LLV), which is associated with persistent low-grade inflammation, fibrosis progression, and increased risk of hepatocellular carcinoma. We aimed to investigate the high-risk factors related to LLV.

**Methods:**

In this retrospective study, patients receiving entecavir (ETV) treatment from January 2018 to January 2023 were enrolled, and were divided into a LLV (HBV DNA 20-2000 IU/mL) cohort and a complete virological response (CVR) (HBV DNA < 20 IU/mL) cohort according to the virological response at week 48 posttreatment. Treatment baseline characteristics were retrieved from electronic medical records. Multivariate logistic regression was performed.

**Results:**

Totally, 1653 patients were enrolled, male patients accounted for 73.0%; the median age was 44 years; the mean HBV DNA level was 5.9 Log_10_ IU/ml. Among them, 472 (28.6%) experienced LLV. Multivariate analysis showed that HBeAg positivity (OR = 2.650, 95% CI: 2.000-3.511, *p* < 0.001), HBV DNA ≥ 6.0 Log_10_ IU/mL (OR = 1.370, 95% CI: 1.054-1.780, *p* = 0.019), qHBsAg ≥ 9000 IU/mL (OR = 4.472, 95% CI: 3.410-5.866, *p* < 0.001), cirrhosis (OR = 1.650, 95% CI: 1.234-2.207, *P* = 0.001), LSM ≥ 13.0 kPa (OR = 1.644, 95% CI: 1.203-2.246, *p* = 0.002), and PLT < 100×10^9^/L (OR = 1.450, 95% CI: 1.094-1.922, *p* = 0.010) at baseline were related to the development of LLV.

**Conclusions:**

High HBV DNA/HBsAg quantification/LSM, low PLT, HBeAg positivity, and liver cirrhosis were high-risk factors associated with LLV in patients receiving entecavir treatment.

## Introduction

Hepatitis B virus (HBV) has currently infected approximately 296 million patients worldwide. Without treatment, chronic HBV infection can cause progressive liver fibrosis, which may lead to cirrhosis, decompensation, and hepatocellular carcinoma (HCC). About 800,000 people die each year caused by HBV infection (Jeng et al., 2023). At present, most guidelines recommend the goal of treating chronic hepatitis B (CHB) patients is to maximize long-term inhibition of HBV replication, improve inflammation and necrosis of hepatocytes and liver fibrosis, reduce the risk of cirrhosis and HCC, and enhance the quality of life and prolong the survival time of CHB patients.

Currently, antiviral treatment includes nucleos(t)ide analogues (NAs) and peg-interferon (Lok et al., 2016). NAs can suppress HBV replication significantly and have a high safety profile. Entecavir (ETV), tenofovir disoproxil fumarate (TDF), and tenofovir alafenamide fumarate (TAF) are the first-line NAs for CHB patients due to its high antiviral potency and genetic barrier to resistance (Hou et al., 2020).

In recent years, during long-term administration of NAs, patients with persistent or intermittent low-level viraemia (LLV, HBV DNA between 20 and 2000 IU/mL) were very common in clinical practice. ETV is the most widely used first-line agent due to its early market time and large patient base and will be used and maintained by more patients in the future. Although most of the patients achieved complete virological response (CVR) with ETV treatment at week 48 in the real world, some patients still had LLV after long-term ETV treatment (Li et al., 2021; Zhang et al., 2021). In addition, with the gradual improvement of the sensitivity of HBV DNA detection methods, patients who were previously considered to have CVR in clinical practice were also confirmed to have LLV after hypersensitivity HBV DNA detection (Revill et al., 2019).

LLV could promote the drug-resisted mutation (Kim H. J., et al., 2017), progression of liver fibrosis (Sun et al., 2020), and significantly increase the risk of HCC (Ji et al., 2022). Therefore, the research on LLV has become a hot and difficult issue in antiviral therapy. However, there are few studies on the risk factors associated with LLV (Li et al., 2023; Chen et al., 2024). We aimed to investigate the risk factors related to LLV in ETV-treated CHB patients, in order to provide evidence for the timely diagnosis and precise treatment of such patients.

## Patients and methods

### Patients

This is a real-world retrospective study, which consisted of inpatients who received ETV treatment. We enrolled patients who met all of the following inclusion criteria: (a) presented to our hospital between January 2018 to January 2023; (b) aged over 18 years; (c) serum positive for both HBsAg and HBV DNA (Terrault et al., 2018); (d) antiviral treatment naïve; (e) had an indication for antiviral therapy (elevated ALT, and/or at least moderate histological lesions by liver biopsy or noninvasive approaches) (EASL, 2017). Patients who met one of the following exclusion criteria were excluded: (a) treated with Peg-IFN-α; (b) coinfected with either hepatitis C virus or human immunodeficiency virus; (c) combined with other etiologies of liver diseases (e.g., autoimmune liver disease (Galaski et al., 2021), drug-induced liver injury (EASL, 2019), etc.); (d) had decompensated cirrhosis; (e) any cancers and (f) women with pregnancy or breastfeeding.

After receiving 0.5mg/d of ETV (Baraclude^®^ [BMS, USA] or Entecavir dispersible tablets^®^ [Suzhou Dongrui, China]) during the research period for 48 weeks, when diagnosing LLV, patients should be ruled out that: (1) the patient has poor compliance with antiviral therapy, such as missing antiviral drugs, self-reducing drug dose, or improper medication methods (such as ETV not followed by fasting at least 2h before or after meals); (2) the possibility of “false positive” results caused by pollution in HBV test.

The study was approved by the Ethics Committees of Fifth Medical Center of PLA General Hospital (No. 2020056D), and conducted in compliance with the 1975 Declaration of Helsinki, Good Clinical Practice guidelines, and local regulatory requirements.

### Measurements

Patients were assessed by physical examinations, blood tests (virological, biochemical parameters), and liver stiffness measurements (LSM) at treatment baseline and every 12 or 24 weeks after treatment. Serum HBV DNA levels were measured by COBAS TaqMan HBV Test (Roche, USA), with 20 IU/mL as the lower limit of detection. HBV serological markers were measured by chemiluminescent immunoassay (Abbott, Germany). Serum quantitative hepatitis B surface antigen (qHBsAg) was measured by chemiluminescent immunoassay using the HBsAg QT assay kit (Roche, USA). For resistance testing, the HBV reverse transcriptase gene was amplified by nested PCR, and then detected by direct population sequencing using an ABI 3730xl DNA Analyzer (Applied Biosystems, USA). The estimated glomerular filtration rate (eGFR) was determined by the Cockcroft-Gault method. Cirrhosis was diagnosed with ultrasound. LSM was performed by experienced operators using transient elastography (FibroTouch^®^; HISKY, China). Subclinical cirrhosis (SCC) was defined as nonclinical cirrhosis but with an LSM ≥ 13.0 kPa (Kim et al., 2015). At treatment baseline, any patient should be determined whether there was a disease such as diabetes mellitus (ADA, 2012), hypertension (Williams et al., 2018), and fatty liver (EASL et al., 2016). Drinking was defined as alcohol intake > 25g/day for men and 15g/day for women lasting at least 5 years (O'Connor et al., 2018), smoking was defined as more than 1 cigarette per day for 6 consecutive or cumulative months (Verbiest et al., 2017).

### Outcomes

According to virologic response at the end of 48-week antiviral therapy, the enrolled patients were divided into a LLV (defined as HBV DNA level still detectable, but < 2000 IU/mL) cohort and a CVR (defined as HBV DNA level < 20 IU/mL) cohort (Terrault et al., 2018). Characteristics of the patients were collected and multivariate logistic regression was performed to identify the independent factors related to LLV.

### Statistical analysis

Categorical variables were expressed as numbers (percentages) and compared using a chi-squared or Fisher’s exact test. Continuous variables with normal distribution were expressed as mean ± standard deviation (SD) and compared with the t-test, and continuous variables with skewed distribution were expressed as median (interquartile range, IQR) and compared with the Mann-Whitney test. Univariate analyses were performed to identify variables that were significantly different between LLV and CVR cohorts. According to the calculated OR and 95% confidence interval (CI), a multivariable logistic regression was further performed to select and eliminate variables. A *p-value* < 0.05 was considered significant for all statistical tests. All the statistical analyses were performed using R software.

## Results

### Baseline characteristics

**Figure 1:**
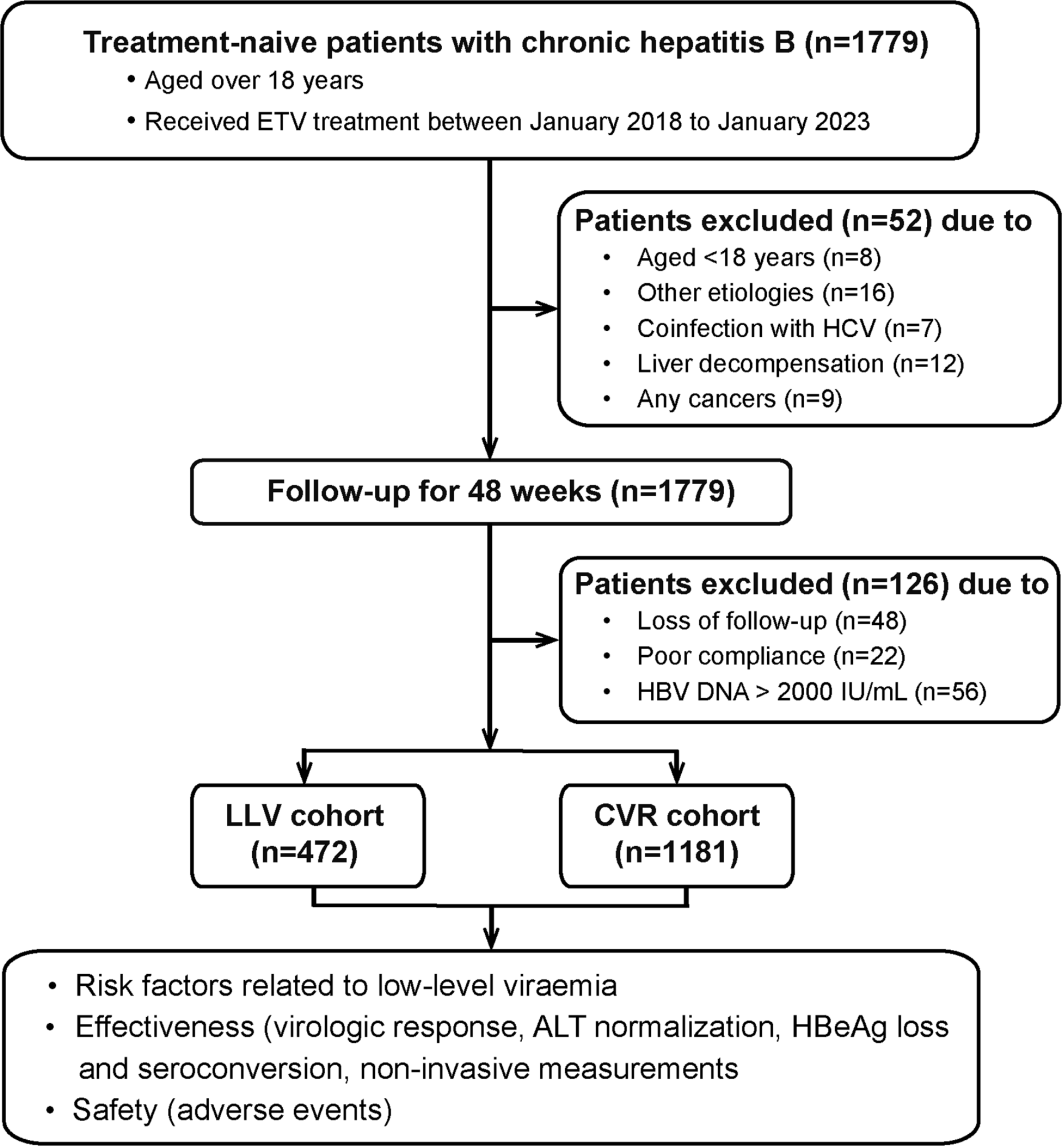
Flow chart of the study population. ETV, entecavir; LLV, low-level viremia; CVR, Complete virological response.

Totally, 1831 patients were screened and 1653 patients were enrolled (**Figure 1**). HBV pol/RT mutation detection was performed in 9.9% (164/1653) patients by sequencing, and no mutation was detected. The percent of male patients was 73.0% (1206/1653) and the median age was 44 years, the mean HBV DNA level was 5.9 Log_10_ IU/mL and the median ALT at baseline was 51 U/L. The median serum qHBsAg was 7647 IU/m. Serum HBeAg was positive in 52.6% (870/1653) patients. The median LSM was 9.7 kPa, and 47.5% (785/1653) patients were diagnosed with cirrhosis. Among them, 1181 (71.4%) patients achieved CVR, and 472 (28.6%) patients experienced LLV. Patients who received Baraclude^®^ treatment or liver protection drugs (glycyrrhizin or silybin) in these two groups showed no statistical difference. The details of baseline clinical characteristics are shown in **Table 1**.

**Table 1:** Baseline clinical characteristics of enrolled patients.

	Overall (n = 1653)	CVR (n = 1181)	LLV (n = 472)	*p value*
Male sex, n(%)	1206 (73.0)	856 (72.5)	350 (74.2)	0.529
Age, years	44 (35,55)	43 (37,57)	44 (36,59)	0.100
BMI, kg/m^2^	23.9 ± 3.3	23.9 ± 3.4	24.1 ± 3.4	0.453
High qHBsAg level, n(%) ^①^	403 (24.4)	173 (14.6)	230 (48.7)	<0.001
High HBV DNA level, n(%) ^②^	834 (50.5)	533 (45.1)	301 (63.8)	<0.001
HBeAg positive, n(%)	870 (52.6)	508 (43.0)	362 (76.7)	<0.001
immune tolerance, n(%) ^③^	165 (10.0)	95 (8.0)	70 (14.8)	<0.001
ALT, U/L	51.0 (32.0, 89.0)	49.0 (31.0, 91.0)	54.5 (36.0, 86.0)	0.016
ALT > ULN, n(%)	1046 (63.3)	719 (60.9)	327 (69.3)	0.002
AST, U/L	46.0 (32.0, 72.0)	45.0 (30.0, 72.0)	48.5 (35.0, 74.0)	0.004
AST > ULN, n(%)	985 (59.6)	673 (57.0)	312 (66.1)	0.001
TBIL > ULN, n(%)	592 (35.8)	431 (36.5)	161 (34.1)	0.392
PLT < LLN, n(%)	394 (23.8)	255 (21.6)	139 (29.4)	0.001
LSM	9.7 (7.6, 12.2)	9.4 (7.4, 11.6)	10.4 (8.0, 17.5)	<0.001
LSM ≥ 13.0 kPa, n(%)	391 (23.7)	231 (19.6)	160 (33.9)	<0.001
Family history, n(%)	704 (42.6)	494 (41.8)	210 (44.8)	0.472
Drinker, n(%)	322 (19.5)	212 (17.9)	110 (23.3)	0.136
Diabetes mellitus, n(%)	251 (15.2)	167 (14.1)	84 (17.8)	0.073
Smoke, n(%)	334 (20.2)	233 (19.7)	101 (21.4)	0.691
Hypertension, n(%)	156 (9.4)	113 (9.6)	43 (9.1)	0.846
Cirrhosis, n(%)	785 (47.5)	525 (44.5)	260 (55.1)	<0.001
Fatty liver, n(%)	165 (10.0)	113 (9.6)	52 (11.0)	0.360
Baraclude® treatment, n(%)	468 (28.3)	347 (29.4)	121 (25.6)	0.143
liver protection drugs, n(%)^④^	257 (15.5)	187 (15.8)	70 (14.8)	0.665

BMI, body mass index; ALT, alanine aminotransferase; AST, aspartate aminotransferase; ULN, the upper limit of normal; LLN, the lower limit of ULN; ALP, alkaline phosphatase; GGT, gamma-glutamyl transpeptidase; TBIL, total bilirubin; PLT, platelet; LSM, liver stiffness measurement. PLT LLN, 100×10^9^/L; ALT ULN, 40 U/L; AST ULN, 40 U/L.

^①^ qHBsAg ≥ 9000 IU/mL; ^②^ HBV DNA ≥ 6 Log_10_ IU/mL; ^③^ normal ALT, positive HBeAg and HBV DNA ≥ 6 Log_10_ IU/mL; ^④^ glycyrrhizin or silybin.

### Virological response

The percentage of patients harboring high viral load (HBV DNA ≥ 6.0 Log_10_ IU/mL, defined by its median value) was 63.8% (301/472) in the LLV cohort, which was significantly higher than 45.1% (533/1181) in the CVR cohort (*p* < 0.001) (**Table 1**). The mean level of HBV DNA at baseline was 5.7 Log_10_ IU/mL in the CVR cohort and undetectable at week 48. The mean level of HBV DNA at baseline was 6.5 Log_10_ IU/mL in the LLV cohort and 2.94 Log_10_ IU/mL at week 48, which was statistically significant (*p* < 0.001). The mean decrease in HBV DNA level was significantly higher in the LLV cohort compared with the CVR cohort (6.5 vs 5.7 Log_10_ IU/mL, *p* < 0.001) (**Table 2**).

**Table 2:** Effectiveness assessment at week 48.

	Week 48		
	CVR (n = 1181)	LLV (n = 472)	*p* value
HBV DNA decrease, Log_10_ IU/mL	5.7 ± 1.8	6.5 ± 0.7	<0.001
HBeAg loss, n/N(%) ^①^	173/508 (34.1)	70/362 (19.3)	0.026
HBeAg seroconversion, n/N(%)	56/508 (11.0)	30/362 (8.3)	0.329
Normalized ALT, n/N(%)^②^	638/719 (88.7)	286/327 (87.5)	0.453
qHBsAg decrease, IU/mL	1,379 (808, 1577)	1,498 (891, 1780)	0.079
HBsAg loss, n(%)	0	0	>0.999
LSM, kPa	7.8 (4.7, 9.2)	9.0 (6.2, 10.7)	0.036

AASLD, American Association for the Study of Liver Diseases; ALT, alanine aminotransferase; HBV, hepatitis B virus; TAF, tenofovir alafenamide; ETV, entecavir.

^①^ Among patients who were seropositive for HBeAg and negative for anti-HBe at baseline.

^②^ Among patients with ALT at baseline above the central lab criteria (≤ 40 U/L).

### HBV biomarker change

The median level of qHBsAg was 7828 (7646) IU/mL at baseline and 6330 (6267) IU/mL at week 48 in the LLV (CVR) cohort. The median level of qHBsAg at baseline between the two cohorts was of statistical significance (*p* < 0.001). The median decrease in qHBsAg level was 1498 IU/mL in the LLV cohort, which showed no significant difference compared with the CVR cohort (1,379 IU/mL, *p* = 0.079). None of the patients showed complete disappearance of the serum HBsAg during the 48-week treatment period. Furthermore, the percentage of patients harboring high qHBsAg (≥ 9000 IU/mL, defined by receiver operating characteristic curve, **Supplementary Figure 1**) was 48.7% (230/472) in the LLV cohort, which was significantly higher than 14.6% (173/1181) in the CVR cohort (*p* < 0.001) (**Table 1**).

In addition, there were 76.7% (362/472) HBeAg-positive patients in the LLV cohort, which was significantly higher than 43.0% (508/1181) in the CVR cohort (*p* < 0.001). The rate of HBeAg loss among HBeAg-positive patients in the LLV cohort was 19.3% (70/362), which was significantly lower than 34.1% (173/508) in the CVR cohort (*p* = 0.026). The rate of HBeAg seroconversion was numerically higher in the CVR cohort, but the difference did not achieve statistical significance (11.0% vs 8.3%, *p* = 0.329) (**Table 2**).

### Changes in ALT and LSM

The median level of ALT at baseline between the LLV and CVR cohorts was of statistical significance (54.5 U/L vs 49.0 U/L, *p* = 0.016). At week 48, under our hospital central laboratory criteria (≤ 40 U/L), the percentage of ALT normalization was 87.5% (286/327) in the LLV cohort, which showed no difference compared with 88.7% (638/719) in the CVR cohort (*p* = 0.453). The median LSM at baseline [week 48] was 10.4 [9.0] kPa in the LLV cohort, which was significantly higher than 9.4 [7.8] kPa in the CVR cohort (*p* < 0.001, [*p* = 0.036], **Table 2**).

### Risk factors related to low-level viraemia

**Figure 2:**
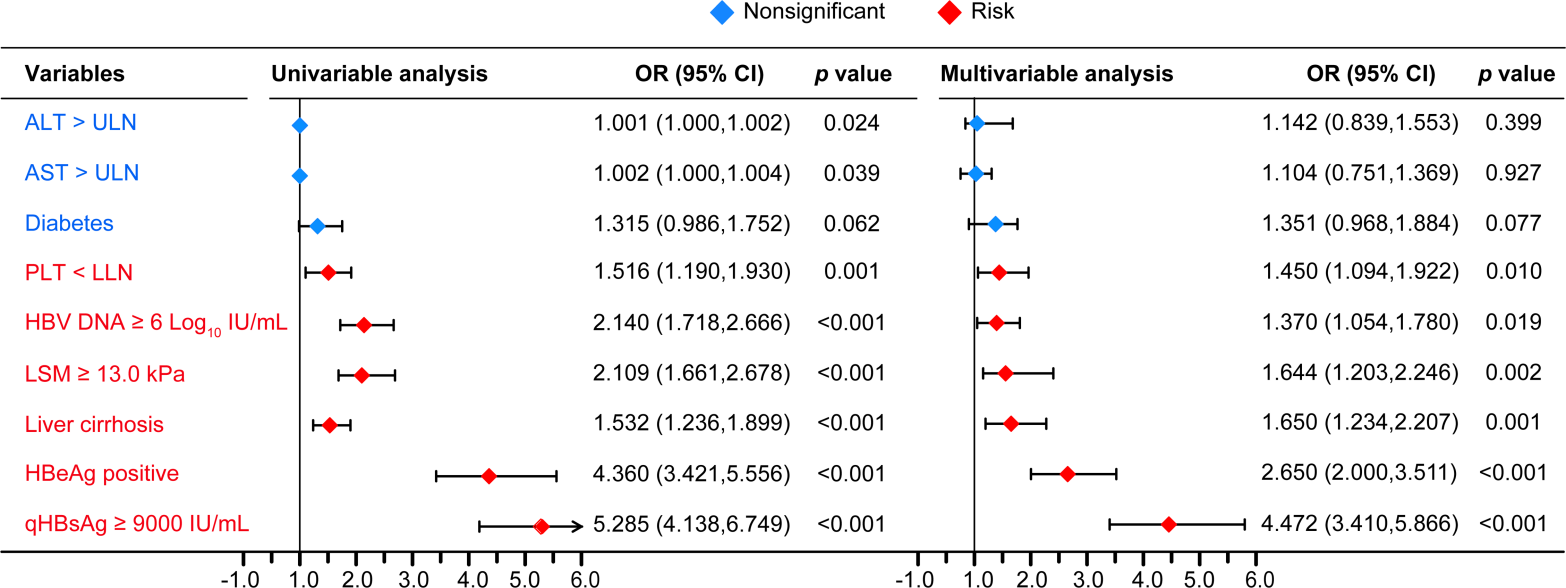
The univariable and multivariable logistic regression analyses. PLT, platelet; LLN, lower limit of ULN; ULN, upper limit of normal; OR, odd ratio; CI, confidence interval. PLT LLN, 100×10^9^/L; ALT ULN, 40 U/L; AST ULN, 40 U/L.

**Figure 3:**
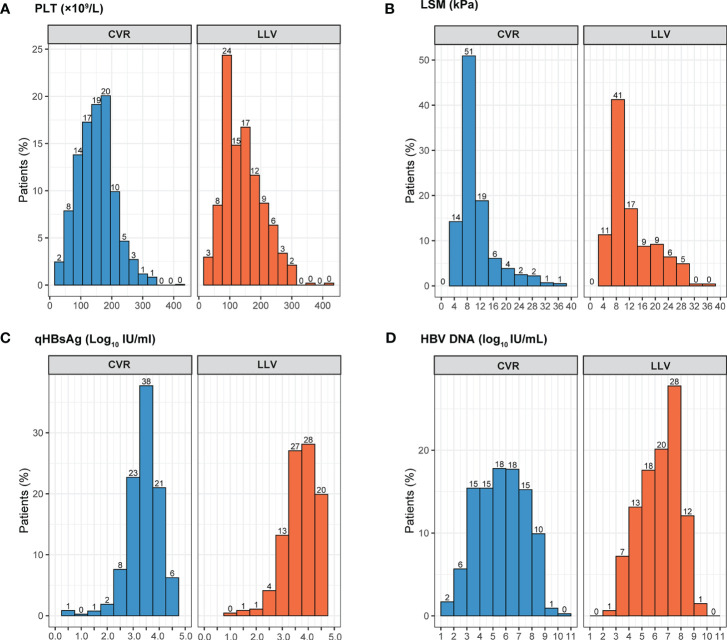
The distributions of the quantitative variables related to LLV by virological response. LLV, low-level viremia; CVR, Complete virological response; PLT, platelet; LSM, liver stiffness measurement. **(A)** PLT distribution by virological response; **(B)** LSM distribution by virological response; **(C)** qHBsAg distribution by virological response; **(D)** HBV DNA distribution by virological response.

After comparison between groups of CVR and LLV, high qHBsAg, high HBV DNA, HBeAg positive, ALT > ULN, AST > ULN, TBIL > LLN, PLT < LLN, cirrhosis, and high LSM were included in the following univariate and multivariate logistic analysis. Whereas, age, sex, family history of HBV infection, alcohol drinking, smoking, diabetes mellitus, hypertension, drug brand, and administration of liver protective drugs showed no impact on the development of LLV. Multivariate logistic regression analysis showed that HBeAg positivity (OR=2.650, 95% CI: 2.000-3.511, *p* < 0.001), HBV DNA ≥ 6.0 Log_10_ IU/mL (OR=1.370, 95% CI: 1.054-1.780, *p* = 0.019), qHBsAg ≥ 9000 IU/mL (OR=4.472, 95% CI: 3.410-5.866, *p* < 0.001), cirrhosis (OR=1.650, 95% CI: 1.234-2.207, *p* = 0.001), PLT < 100×10^9^/L (OR = 1.450, 95% CI: 1.094-1.922, *p* = 0.010), and LSM ≥ 13.0 kPa (OR=1.644, 95% CI: 1.203-2.246, *p* = 0.002) at baseline were independent high-risk factors related to LLV in CHB patients after long-term ETV treatment (**Figure 2**). The distributions of the above-mentioned quantitative variables are shown in **Figure 3**.

**Figure 4:**
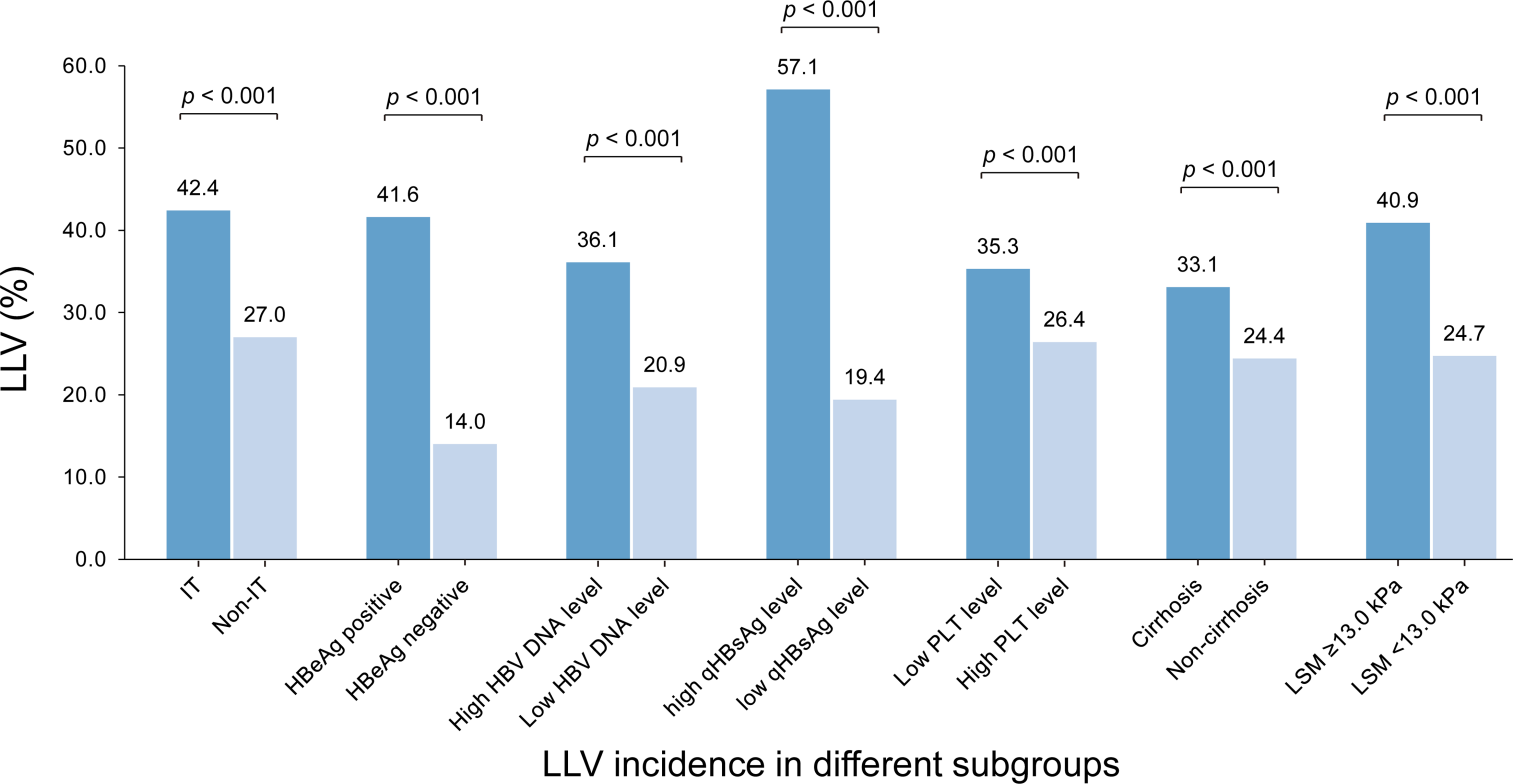
The incidences of LLV in different subgroups by the identified high-risk factors. IT, immune tolerance (defined as normal ALT, positive HBeAg and HBV DNA ≥ 6 Log_10_ IU/mL); LLV, low-level viremia; PLT, platelet; LSM, liver stiffness measurement.

Among 10.0% (165/1653) of patients with immune tolerance (IT), defined as HBeAg positive, normal ALT and high HBV DNA (≥ 6 Log_10_ IU/mL), the LLV incidence was 42.4% (70/165), which was significantly higher than 27.0% (402/1488) in non-IT subgroup (p < 0.001); Among 52.6% (870/1653) of patients with positive HBeAg, the LLV incidence was 41.6% (362/870), which was significantly higher than 14.0% (110/783) in HBeAg negative subgroup (p < 0.001). The incidence of LLV in other subgroups is shown in **Figure 4**.

## Discussion

Antiviral therapy is of fundamental strategy to prevent disease progression in CHB patients. ETV, as a clinically recommended first-line agent, can rapidly suppress HBV DNA replication and improve the progression of virology, histology, biochemistry, and related complications (Martinez et al., 2021).

Despite the widespread use of NAs, 20%-37% of patients still experienced LLV. The reason may be related to the limitation of NAs’ competitive inhibition of HBV replication and the mechanism of no direct effect on cccDNA, which exists stably in the nucleus of infected hepatocytes in the form of tiny chromosomes (Allweiss and Dandri, 2017). EASL guidelines have summarized the HBV DNA response in patients after ETV treatment: after 48 or 52 weeks of ETV treatment, the detectable rate of HBV DNA in HBeAg-positive or negative patients was 33% or 10% respectively (HBV DNA < 60~80 IU/mL) (EASL, 2017). With the continuous innovation of HBV DNA quantitative detection technology, the detection limit of viral load is getting lower and lower. Thus, the virological response defined by the older test may be classified as LLV when more sensitive tests are used. In a retrospective analysis of 894 CHB patients receiving ETV, 240 cases (26%) experienced LLV (HBV DNA > 12 IU/mL) during a follow-up period of 5 years (Kim et al., 2018). These results suggest that the incidence of LLV may be underestimated. In our study, the proportion of patients with LLV was up to 28%, which was consistent with previous studies. The level of HBV DNA is the most direct indicator of HBV replication and infectivity in CHB patients. The lower the HBV DNA level in CHB patients receiving antiviral therapy, the higher the probability of early virological response and HBeAg seroconversion. Studies have shown that the lower the pre-treatment HBV DNA level in patients treated with ETV, the more likely they are to achieve a CVR (Yuen et al., 2011). Corresponding point of view, studies reported that HBV DNA ≥10^8^ IU/mL at baseline significantly influenced the efficacy of NAs at 48 weeks, with a 33% rate of HBV DNA detectable at 52 weeks and 23% at 3 years (Lok et al., 2012). These patients with high viral load may be advised to prolong treatment and still have LLV. Kim et al. found that baseline HBV DNA load before treatment was an important risk factor for LLV (Kim et al., 2019). The results of our study also identified that HBV DNA ≥ 6 Log_10_ IU/mL at baseline was an independent risk factor for LLV, which was similar to the literature above.

HBV DNA levels tend to be higher in HBeAg-positive CHB patients than in HBeAg-negative patients, and the rate of HBV DNA undetectable is lower after antiviral treatment. Studies reported that HBeAg-positive patients have a higher risk of drug resistance, and are prone to virological breakthrough or poor virological response during long-term antiviral therapy (Wong et al., 2012). Few research has been conducted on the effect of baseline HBeAg status on the LLV during antiviral therapy. Our study showed that HBeAg-positive patients in the CVR cohort or the LLV cohort accounted for 43% or 77%, respectively. Therefore, based on the analysis, the rate of HBeAg positive at baseline was an independent risk factor for LLV.

The quantitative level of HBsAg reflects the HBV DNA replication, cccDNA transcription, and the rate of HCC incidence. Studies reported that HBsAg quantification before antiviral therapy is related to virological response, and HBsAg/HBV DNA > 0.56 can predict the virological response in CHB patients (Song et al., 2011). Other studies showed that the lower the HBsAg quantification at baseline, the easier it is to obtain a CVR (Wong et al., 2022). However, few studies have analyzed the correlation between HBsAg quantification and the risk of LLV. The results of our study showed that HBsAg quantification at baseline was significantly higher in the LLV cohort than in the CVR cohort and that qHBsAg ≥ 9000 IU/mL was an independent risk factor for LLV.

In addition, we identified that the higher the degree of liver fibrosis (low PLT, liver cirrhosis, even subclinical cirrhosis), the higher the risk of LLV. It meant that those patients with higher degrees of liver fibrosis should choose more powerful antiviral drugs and timely adjust their antiviral strategy once LLV was detected. The corresponding III phase RCT revealed that the 48-week incidence of detectable HBV DNA in HBeAg-positive patients receiving ETV (cutoff: 300 copies/mL), TDF (cutoff: 29 IU/mL), or TAF (cutoff: 29 IU/mL) treatment was 24%, 36%, and 33%, respectively (Gish et al., 2007; Agarwal et al., 2018). A retrospective study including 1042 patients reported that the risk of LLV was lower in the TAF-treated patients compared with ETV-treated patients (adjusted OR=0.50,95% CI 0.26-0.96, *p* = 0.040) (Wang et al., 2022). However, we cannot deduce which drug is better due to the lack of prospective head-to-head clinical studies so far. Moreover, for those with higher degrees of liver fibrosis, when (week 24 or week 48) to adjust their treatment is still unknown and needs further research to determine.

Furthermore, we also found that the incidence of LLV in patients with IT was higher than that in its counterpart subgroup. It has already proved that a portion of patients in the IT stage had obvious liver fibrosis and poor prognosis, active antiviral therapy should be carried out for long-term benefit (Liu et al., 2021; Huang et al., 2022). Due to the results of the present study, antiviral therapy in these patients was of particular concern in choosing more effective NA regimen, or considering the combination of two NA regimens.

Some research focused on the risk factors of LLV, we analyzed their study design and conclusions. Li J et al. presented that HBV DNA level ≥8 Log_10_ IU/mL, anti-HBc level < 3 Log_10_ IU/mL, and HBeAg seropositivity at baseline contribute to LLV in patients with CHB receiving 78-week antiviral treatment (Li et al., 2023). Chen H et al. showed that CHB patients with a high HBV DNA load, high HBsAg quantification, and positive HBeAg results tend to have a high risk of LLV despite long-term ETV antiviral treatment (Chen et al., 2024). The above studies seemed similar to our study, but their enrolled patients were small, neither study had more than 400 cases, but our analysis included 1653 cases, and the results were more reliable. Furthermore, our risk factors were more detailed and stratified, which is more convenient and reasonable for clinical application.

Most studies indicated that LLV was not benign and associated with a worse clinical outcome, such as persistent low-grade inflammation, liver fibrosis progression, and increased risk of HCC (Kim J. H., et al., 2017; Kim et al., 2019; Sun et al., 2020; Ji et al., 2022). At present, more prospective data on the hazards of LLV are lacking. However, the prognosis of patients with LLV treated with NAs deserves more attention. Some studies focus on different rescue strategies, such as under prolonged entecavir monotherapy, switching to or in combination with TDF, switching to TAF, or in combination with PEG-IFN-α (Uchida et al., 2020; Yin et al., 2021). EASL guidelines indicate that any NAs are likely to develop LLV. Most patients with high HBV DNA levels are more likely to experience LLV after ETV or TDF treatment, which is related not to drug inefficacy but to their limited ability to inhibit viral replication. Therefore, pharmacokinetics should be considered for patients with high HBV DNA levels at 48 weeks when they experienced LLV: prolonged treatment may increase the response rate for those with a continuous decline in HBV DNA level, and the risk of long-term drug resistance is very low, so original antiviral monotherapy can be continued. Another NAs can be substituted or added if HBV DNA does not decline. Peg-IFN-α has more advantages than NAs in HBeAg seroconversion and HBsAg clearance. However, the relatively weak antiviral effect, low tolerance, and high risk of adverse events limit its use. Therefore, how to screen patients with a good response has become a key issue regarding PEG-IFN-α treatment. Studies have shown that for HBeAg-positive patients with poor response to NAs, further combined treatment with PEG-IFN-α can significantly inhibit HBV DNA replication (Wang et al., 2023) (**Supplementary Figure 2**). If the research results can be verified in a large clinical cohort, it will undoubtedly provide new ideas and methods for the rescue treatment of LLV.

Several limitations should be noted. Firstly, the retrospective study design had its inherent disadvantages, which need to be verified in the prospective cohort. However, our sample size was relatively bigger with less bias, compared with previously published articles. Secondly, other NAs except ETV were not analyzed in the current study, making it impossible to compare the potential difference among different NAs. So, the conclusion should only apply to patients receiving ETV treatment. Nevertheless, this study design can reduce the selection bias, because the first line NAs were not approved at the same time. So far, ETV-treated patients remained the biggest population compared with other NAs, making the current study more clinical meaningfulness. Lastly, the current study did not include those with HBV DNA > 2000 IU/mL after 48-week treatment, who accounted for only 3.1% (56/1779) of the screened population. We excluded them to facilitate the analysis (two group comparison). On the other hand, the incidence of LLV is over 20%, which is more clinically significant than 3.1% of non-responders or sub-optional response, whom are less mentioned recently due to the high efficacy of NAs.

Our study indicates that high HBV DNA level, high HBsAg quantification, HBeAg positivity, cirrhosis, higher LSM value, and lower PLT level were high-risk factors associated with LLV. With the application of more sensitive HBV DNA detection reagents and methods, more LLV will be detected in clinical practice. In addition to the inherent defects of NAs, are there any other factors affecting the pathogenesis of LLV, such as host immunity, genetics, regulation of hepatocyte proliferation, or other aspects? For the clinical outcome of LLV, do different HBV DNA levels have different effects on disease progression and prognosis? How to optimize the existing rescue treatment strategy until new drugs are successfully developed to cure HBV? These problems need to be further studied and solved to reveal the mystery between HBV DNA replication and clearance.

## Data availability statement

The raw data supporting the conclusions of this article will be made available by the authors, without undue reservation.

## Ethics statement

The studies involving humans were approved by the Ethics Committees of Fifth Medical Center of PLA General Hospital. The studies were conducted in accordance with the local legislation and institutional requirements. The ethics committee/institutional review board waived the requirement of written informed consent for participation from the participants or the participants’ legal guardians/next of kin because the retrospective study design.

## Author contributions

Z-BL: Data curation, Formal analysis, Investigation, Methodology, Writing – original draft, Writing – review & editing. D-DC: Data curation, Investigation, Writing – original draft. Y-FJ: Investigation, Validation, Writing – original draft. Q-JH: Investigation, Validation, Writing – original draft. LC: Investigation, Writing – original draft. F-XD: Investigation, Writing – review & editing. Y-JK: Investigation, Writing – review & editing. XF: Investigation, Writing – original draft. MH: Data curation, Investigation, Methodology, Writing – original draft. X-YJ: Data curation, Investigation, Writing – original draft. JC: Data curation, Methodology, Writing – review & editing. YW: Investigation, Writing – review & editing. DJ: Writing – original draft, Writing – review & editing. GL: Writing – review & editing. S-GW: Writing – review & editing.
