# Supplementary figures and images for: Risk factors related to low-level viraemia in chronic hepatitis B patients receiving entecavir treatment
Source: Front Cell Infect Microbiol. 2024 Aug 7;14:1413589. doi: 10.3389/fcimb.2024.1413589 (PMC11335720; doi:10.3389/fcimb.2024.1413589)

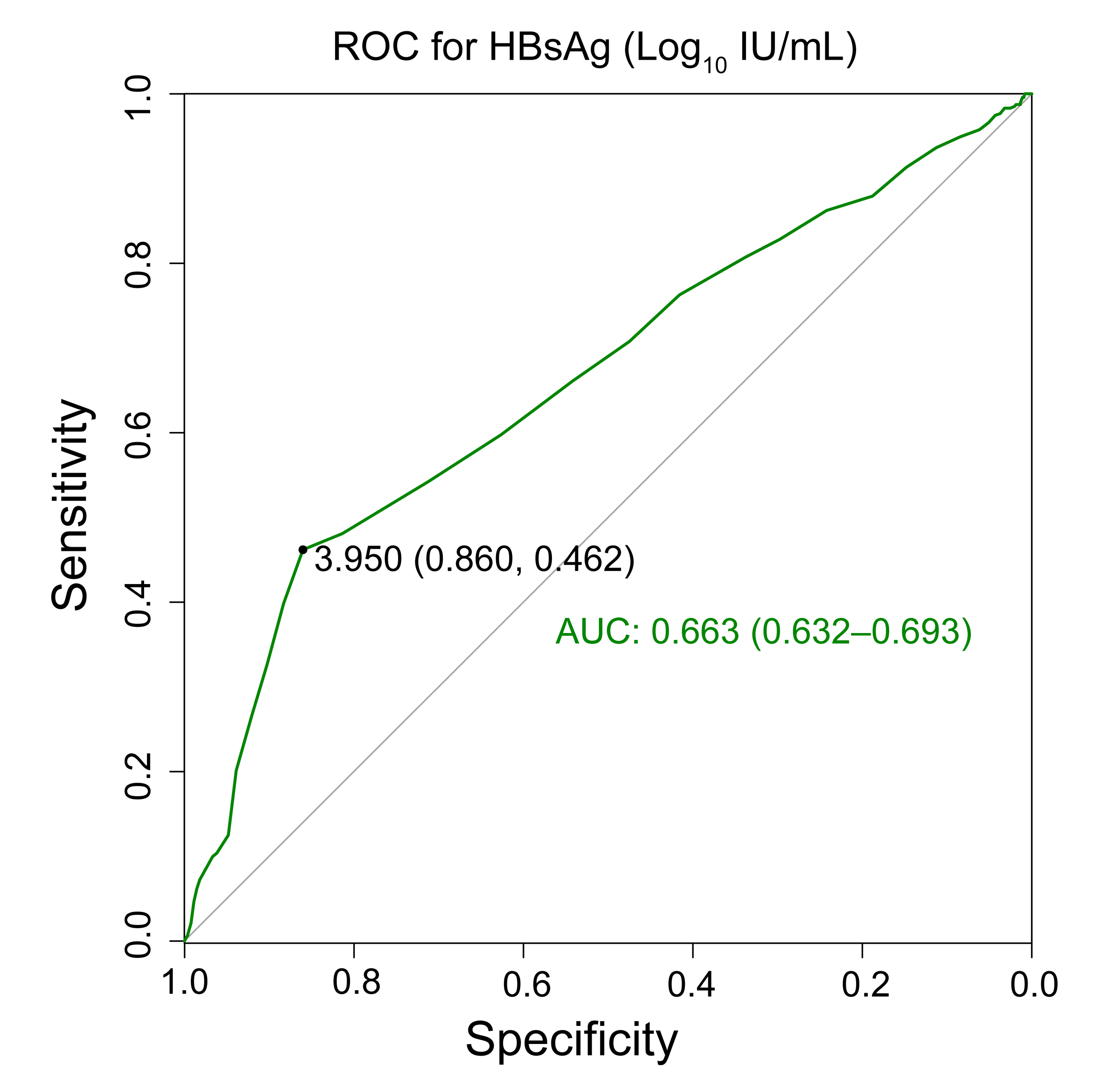

Supplement: Supplementary file 1 [file Image_1.tif]

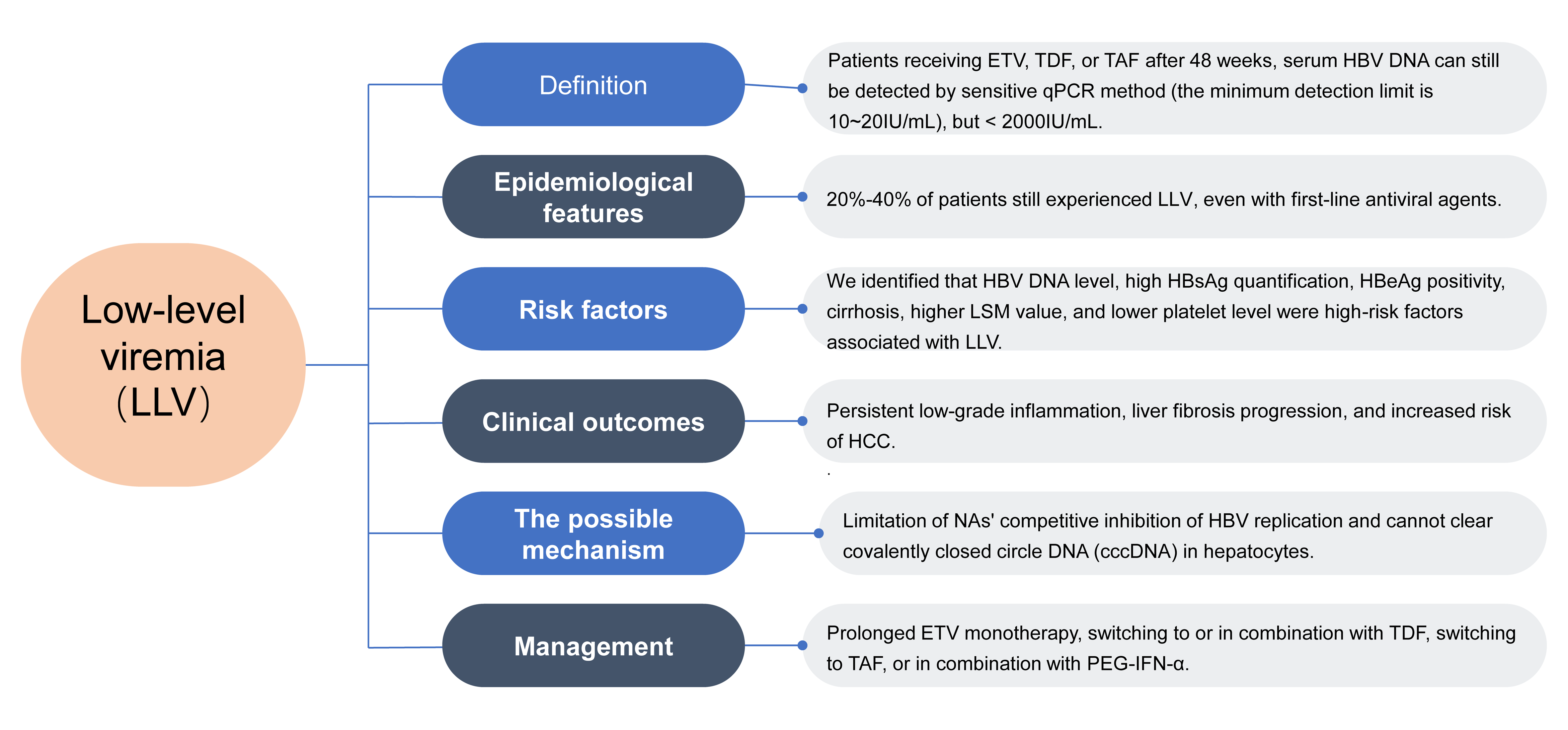

Supplement: Supplementary file 2 [file Image_2.tif]
